# Brain perfusion changes in beta-thalassemia

**DOI:** 10.1186/s13023-024-03194-x

**Published:** 2024-05-21

**Authors:** Renzo Manara, Sara Ponticorvo, Marcella Contieri, Antonietta Canna, Andrea Gerardo Russo, Maria Cristina Fedele, Maria Chiara Rocco, Adriana Borriello, Silvia Valeggia, Maria Pennisi, Marianna De Angelis, Domenico Roberti, Mario Cirillo, Francesco di Salle, Silverio Perrotta, Fabrizio Esposito, Immacolata Tartaglione

**Affiliations:** 1 Neuroradiology, Department of Neuroscience, University of Padua, Padua, Italy; 2 Dipartimento di Medicina e Chirurgia, Scuola Medica Salernitana, Università di Salerno, Fisciano, Italy; 3 Center for Magnetic Resonance Research (CMRR), Department of Radiology, University of Minnesota, 2021 6th St. SE, Minneapolis, MN 55455 USA; 4 Department of Radiology, Center for Magnetic Resonance Research, University of Minnesota, Minneapolis, USA; 5 Department of Advanced Medical and Surgical Sciences, University of Campania “Luigi Vanvitelli”, Napoli, Italy; 6 Dipartimento della Donna, del Bambino e di Chirurgia generale e specialistica, Universit? degli Studi della Campania “Luigi Vanvitelli”, Via Luigi de Crecchio 4, Napoli, 80138 Italy; 7 Pediatrics and Residency Program of Pediatrics, Department of Medicine, Surgery and Dentistry “Scuola Medica Salernitana”, University of Salerno, Baronissi, 84081 Italy; 8 Department of Precision Medicine, University of Campania “L. Vanvitelli”, Via Luigi de Crecchio 7, Naples, Italy

**Keywords:** Brain, Perfusion, Thalassemia, Hemoglobin, Transfusions

## Abstract

**Background:**

Brain injury in hereditary hemoglobinopathies is commonly attributed to anemia-related relative hypoperfusion in terms of impaired oxygen blood supply. Supratentorial and infratentorial vascular watershed regions seem to be especially vulnerable, but data are very scarce.

**Aims:**

We investigated a large beta-thalassemia sample with arterial spin labeling in order to characterize regional perfusion changes and their correlation with phenotype and anemia severity.

**Methods:**

We performed a multicenter single-scanner cross-sectional 3T-MRI study analyzing non-invasively the brain perfusion in 54 transfusion-dependent thalassemia (TDT), 23 non-transfusion-dependent thalassemia (NTDT) patients and 56 Healthy Controls (HC). Age, hemoglobin levels, and cognitive functioning were recorded.

**Results:**

Both TDT and NTDT patients showed globally increased brain perfusion values compared to healthy controls, while no difference was found between patient subgroups. Using age and sex as covariates and scaling the perfusion maps for the global cerebral blood flow, beta-thalassemia patients showed relative hyperperfusion in supratentorial/infratentorial watershed regions. Perfusion changes correlated with hemoglobin levels (*p* = 0.013) and were not observed in the less severely anemic patients (hemoglobin level > 9.5 g/dL). In the hyperperfused regions, white matter density was significantly decreased (*p* = 0.0003) in both patient subgroups vs. HC. In NTDT, white matter density changes correlated inversely with full-scale Intelligence Quotient (*p* = 0.007) while in TDT no correlation was found.

**Conclusion:**

Relative hyperperfusion of watershed territories represents a hemodynamic hallmark of beta-thalassemia anemia challenging previous hypotheses of brain injury in hereditary anemias. A careful management of anemia severity might be crucial for preventing structural white matter changes and subsequent long-term cognitive impairment.

## Introduction

Beta-thalassemia is a rare congenital anemia caused by defective production in the beta-globin chain of hemoglobin. The clinical phenotype is rather heterogeneous. The most severe forms require regular blood transfusions from the first years of life to survive until adulthood (Transfusion Dependent Thalassemia, TDT), while less severe patients receive transfusions occasionally, e.g., during infections or pregnancy (Non Transfusion Dependent Thalassemia, NTDT). In regularly transfused patients, the target is to keep pre-transfusion hemoglobin level at 9.0–9.5 g/dL, to balance the risks of hypertransfusion (e.g., excessive iron overload) and chronic anemia [1]. In general, the management of disease- or treatment-related complications is complex and multisystem deterioration usually occurs in spite of careful laboratory and clinical monitoring. Besides heart, liver and endocrine glands, the brain is another known target of disease complications and cognitive functioning has been shown repeatedly to be impaired, especially among adult TDT patients [1]. However, the precise pathogenesis of brain involvement still remains unclear. Iron overload does not seem to directly affect the neural tissue as iron does not seem to cross the blood-brain barrier in spite of very high serum levels [2–4]. White matter microvascular impairment is also questionable, at least among patients treated according to the current guidelines [5, 6]. Intracranial vasculature (arteries and venous sinuses) does not appear to be significantly involved as increased rate or evolution of intracranial stenosis, venous thrombosis, and aneurysms were not confirmed in recent literature [5, 7]. Recently, a regionally specific vulnerability of the white matter was shown in a large sample of anemias, including beta-thalassemia [8]. In particular, decreased white matter volume and microstructural integrity were found in the watershed areas, i.e. the white matter regions that are in between two main cerebral artery territories [8, 9], suggesting an increased regional perfusion vulnerability of the brain. For these reasons, we performed a multicenter single-scanner cross-sectional MRI study analyzing non-invasively in beta-thalassemia patients and healthy controls the brain perfusion changes occurring in relation with clinical phenotype, age, hemoglobin levels, cognitive functioning and parenchymal lesions. The aims of this study were to (i) confirm and characterize perfusion changes in beta-thalassemia according to phenotype, demographic data and laboratory findings and (ii) correlate regional perfusion changes to parenchymal abnormalities and cognitive functioning impairment.

## Materials and methods

### Subjects

Patients with diagnosis of beta-thalassemia and age > 16 years were recruited from 4 referral centers for beta-thalassemia. Patients were considered with TDT if on a regular transfusion regimen; those who were not regularly transfused and had not received any transfusion during the last 48 weeks before the enrolment were enrolled as NTDT patients. Contraindications to MRI, history of neurologic disease, head trauma or neurosurgery were exclusion criteria. A clinical chart review allowed the collection of clinical and laboratory data related to the disease history and severity; laboratory parameters were collected as mean of last year’s values. In TDT patients, hemoglobin value at MRI was calculated according to post-transfusion hemoglobin level and the rate of hemoglobin decrease per day using a well-established formula applied for transfusion planning in beta-thalassemia patients. All data were acquired between June 2016 and June 2017. We recruited 77 beta thalassemia patients (54 TDT and 23 NTDT patients) and 56 healthy controls. The latter group was volunteers aged > 16 years mostly recruited among patients’ relatives and entourage with a negative history for neurological, hematological, neoplastic or systemic disease, including anemia.

Patients and healthy subjects also participated in our previous multimodal MRI studies [1, 3, 5, 6]. The study was approved by the Ethic Committee (161/2015), and all subjects, both patients and controls, signed an Informed Consent before data collection.

### MRI data evaluation

All study participants underwent brain MRI on the same 3T scanner (MAGNETOM Skyra, Siemens, Erlangen Germany) with a 20-channel head coil.

Cerebral Perfusion weighted maps (PWI) were obtained from brain images acquired using a 3D Pulsed Arterial Spin Labeling (PASL) sequence. Imaging parameters and data analysis methodology can be found in our auditory cortex perfusion study [10].

In order to account for the intrinsic inter-subject variability related to both physiological and technical aspects of ASL acquisition, PWI images were considered both before and after value normalization by the global cerebral blood flow (obtaining a relative cerebral flow, rCBF). A global value was calculated for each subject the mean PWI across all the voxels in the brain was calculated and used as a normalization factor. For the voxel-wise analysis, a general linear model (GLM) full factorial design (as implemented in SPM12 package https://www.fil.ion.ucl.ac.uk/spm/) was used (in both analyses) with one between-subject factor (group) of three levels (HC, TDT, NTDT) and two covariates (age and sex). T-maps were thresholded at *p* < 0.001 voxel-level and only clusters at *p* < 0.05 family-wise error corrected at the cluster level were considered significant.

All patients (TDT and NTDT) were additionally subdivided based on hemoglobin levels (9.5 g/dL cut-off level) and the two subgroups were further separately compared to HC in terms of whole brain brain perfusion.

Finally, mean white matter (WM) density in the cluster of significant perfusion difference was also extracted and compared between groups. Particularly, the whole brain maps of WM density were obtained from tissue segmentation using SPM12, and the mean across all the voxels in the cluster mask was extracted for each subject.

### Cognitive functioning assessment

Study subjects underwent a pool of psychometric tests included in the Wechsler Adult Intelligence Scale-Fourth Edition (WAIS-IV) [1]. For this analysis Full-Scale Intelligence Quotient (FSIQ) and WAIS main domain scores were considered .

### Statistical analysis

Comparisons between groups were performed using the T test, the Mann-Whitney U test and the Chi-square test (or the Fisher Exact test when required) for respectively normally distributed, ordinal and qualitative non-ordinal variables. The linear correlation between two variables was tested using Spearman’s correlation. Statistical significance was set at *p* < 0.05. Finally, an analysis of covariance (ANCOVA) was used to assess the linear dependence of the mean rCBF values in the significant clusters and hemoglobin values. The effect of group (HC, TDT and NTDT) on WM density in the cluster of hyperperfusion was compared with a 1-ANOVA and two-sample post-hoc t-tests.

## Results

Patients’ and controls’ main clinical characteristics are summarized in Table 1.

**Table 1:** Patients’s demographics and clinical characteristics

	TDT (*n* = 54)	NTDT (*n* = 23)	All Patients (*n* = 77)	Controls (*n* = 56)
Mean age, years	36.4 ± 9.2	30.3 ± 11.5	34.6 ± 10.5	33.9 ± 10.8
Females, n (%)	34 (62.9)	13 (56%)	47 (61.0)	36 (64.3)
Splenectomized, n (%)	35 (64.8)	9 (39%)	44 (57.1)	0
Hb*(g/dL), mean ± SD	9.2 ± 0.5	9.3 ± 0.9	9.3 ± 0.8	NA
Hb at MRI (g/dL), mean ± SD	9.6 ± 1.1	9.3 ± 0.9	9.5 ± 1	NA
Ferritin (ng/mL), mean ± SD	877.0 ± 684.4	364.1 ± 290.8	736.2 ± 641.7	NA
LIC (mg/gdw), mean ± SD	4.3 ± 3.0	6.7 ± 5.4	4.9 ± 3.7	NA
**Iron Chelation Therapy**				
DFO, n (%)	14 (25.9)	2 (8.7)	16 (20.8)	0
DFP, n (%)	4 (7.4)	0	5 (6.5)	0
DFX, n (%)	35 (64.8)	4 (17.4)	38 (49.3)	0

*mean Hb in TDT refers to pre transfusional values; LIC: liver iron concentration; DFO: Deferoxamine; DFP: Deferiprone; DFX: Deferasirox

**Fig. 1:**
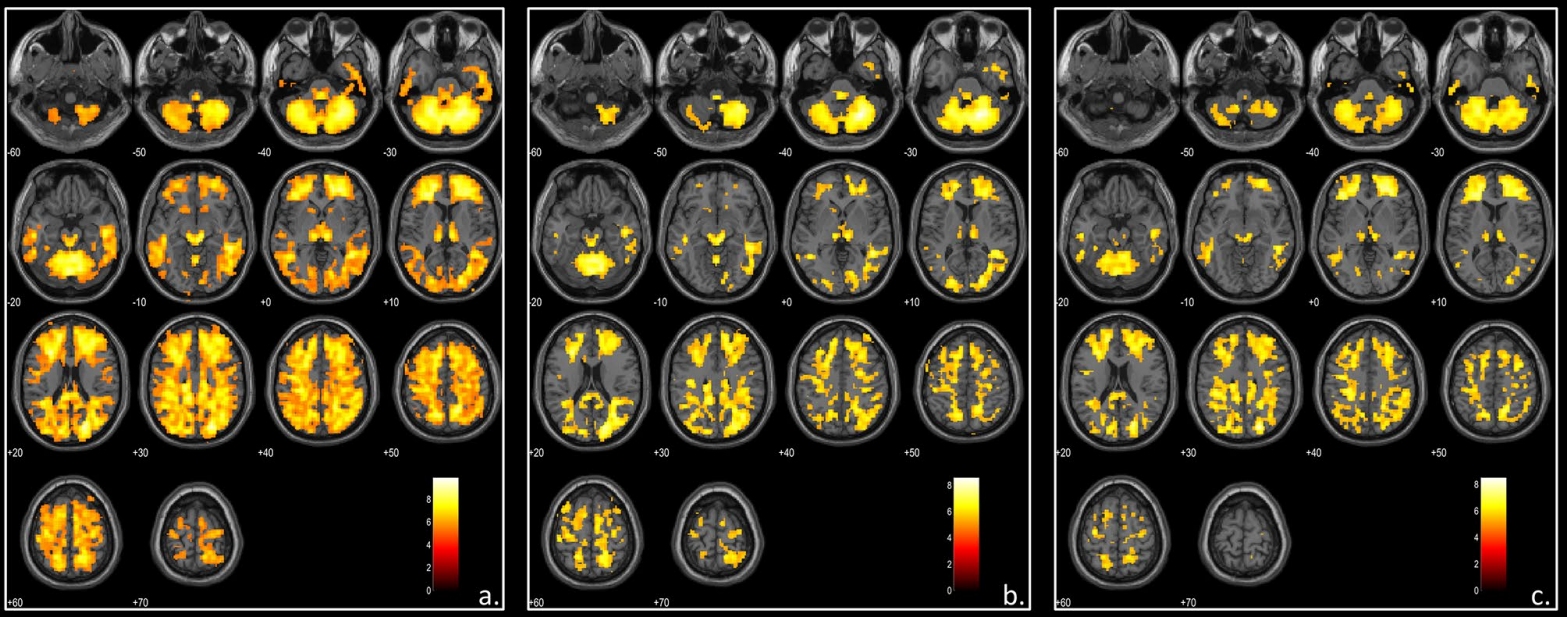
Analysis of brain perfusion without correcting for global cerebral blood flow. T-maps showing significantly globally increased perfusion (colored areas) compared to healthy controls in thalassemia patients (**a**) transfusion dependent thalassemia patients (**b**) and non-transfusion dependent thalassemia patients

Beta-thalassemia patients showed globally increased brain perfusion values compared to HC (Fig-1a); the finding was confirmed also considering patients subgroups (TDT and NTDT; Fig-1b-c), while no difference was found between TDT and NTDT subgroups (data not shown).

**Fig. 2:**
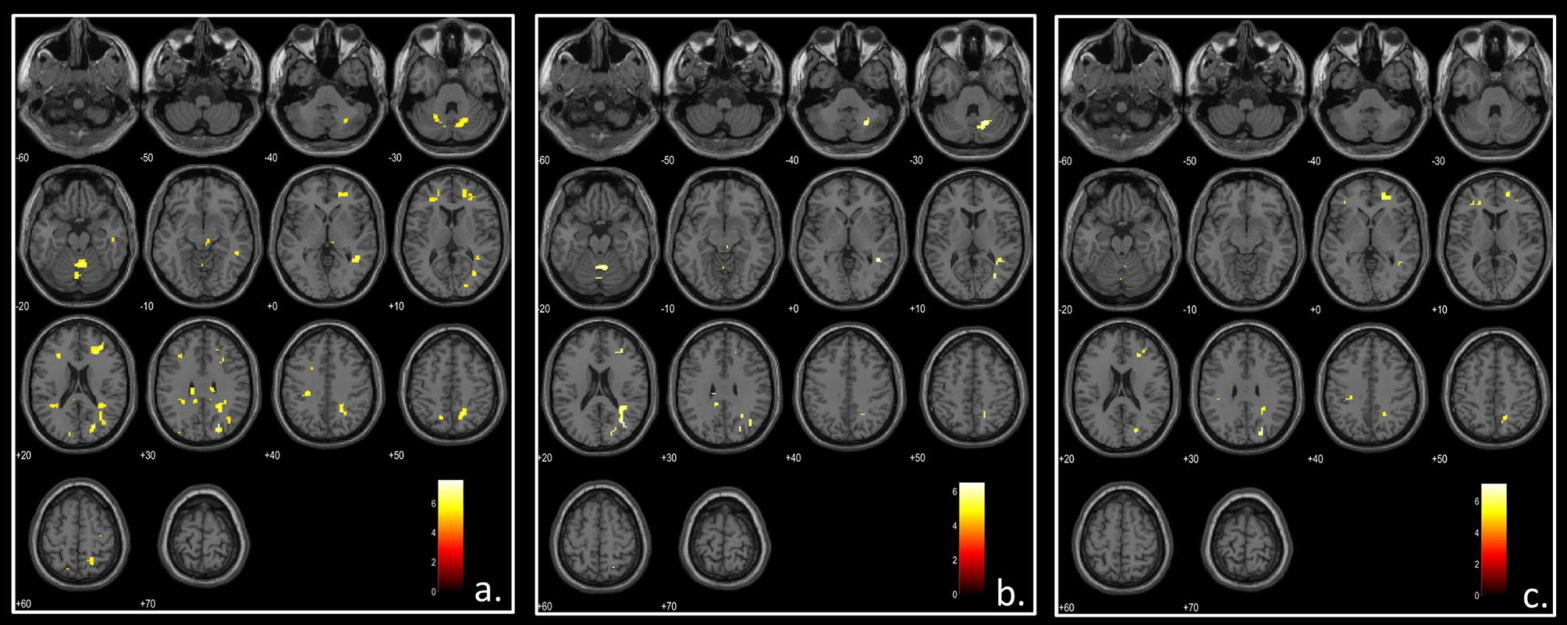
Analysis of brain perfusion correcting for global cerebral blood flow. T-maps showing significantly increased perfusion (colored areas) compared to healthy controls in thalassemia patients (**a**) transfusion dependent thalassemia patients (**b**) and non-transfusion dependent thalassemia patients

Compared to healthy controls, using age and sex as covariates and scaling the perfusion maps for the global CBF, beta-thalassemia patients showed:

1. Several almost symmetric clusters of relative hyperperfusion in the white matter of the centrum semiovale bilaterally, located in the watershed regions between the vascular territories of the main cerebral arteries (namely the anterior, middle and posterior cerebral arteries) (Fig-2a).
2. Bilateral clusters of relative hyperperfusion in the cerebellar white matter corresponding to the cerebellar watershed regions (Fig-2a).

Similar hyperperfusion clusters were obtained comparing HC with patient subgroups (Fig-2b-c); clusters were more evident in the HC-vs-TDT comparison, most likely because of sample size. No significant perfusion differences emerged comparing TDT vs. NTDT or splenectomized vs. non-splenectomized patients.

**Fig. 3:**
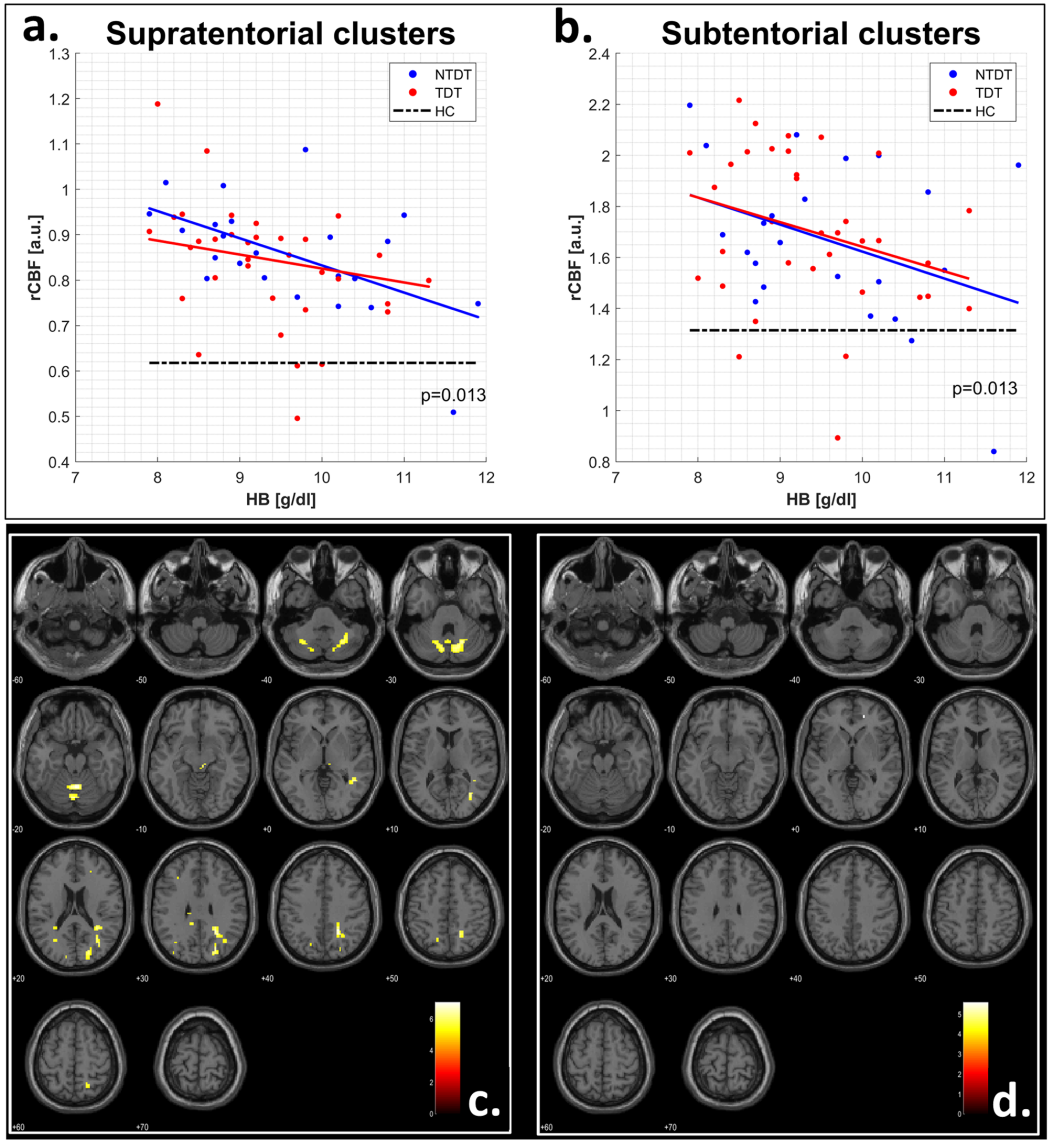
(**A** and **B**) Scatter plots and fitted linear trends of the correlation between regional perfusion values and hemoglobin levels. Blue dots and lines represent non-transfusion thalassemia (NTDT) patients while red dots and lines represent transfusion thalassemia (TDT) patients. Black dashed lines represent mean perfusion values in the same clusters across all healthy controls (HC). (**C** and **D**) Analysis of brain perfusion separating thalassemia patients based on hemoglobin levels (Hb < 9.5 g/dL, panel C; Hb > 9.5 g/dL, panel D)

In patients, both cerebral and cerebellar hyperperfusion clusters correlated with hemoglobin levels (*p* = 0.013) even when subdividing patients according to phenotype severity (Fig-3a-b).

Subdividing patients according to anemia severity (hemoglobin level < or > 9.5 g/dL) the hyperperfusion clusters persisted exclusively in the subgroup with lower hemoglobin levels (Fig-3c-d).

No correlation was found between perfusion values and ferritin, liver iron concentration or chelation treatment.

White matter density in the cluster of perfusion alteration was significantly decreased (*p* = 0.0003, F(df = 2) = 8.83) in both patients subgroups vs. HC (post-hoc t-test: HC vs. TDT *p* = 0.00047; HC vs. NTDT *p* = 0.0049). No significant differences in white matter density were detected while subgrouping patients according to phenotype or anemia severity (*p* > 0.05).

In NTDT, white matter density changes correlated inversely with FSIQ (*p* = 0.007, rho = 0.584), while in TDT no correlation was found (*p* = 0.96, rho = 0.007).

No correlation emerged between hyperperfusion values and full-scale intelligence quotient scores or main WAIS domains (PRI, PSI, VCI and WMI).

## Discussion

The present MRI study investigated brain perfusion in a large sample of beta-thalassemia patients and healthy controls. While confirming the expected compensatory global increase of cerebral blood perfusion secondary to anemia severity (regardless of phenotype), the study also showed in beta thalassemia patients significantly increased relative perfusion in the cerebral and cerebellar watershed territories.

So far, brain tissue injury in anemias has been mostly ascribed to possible chronic impairment of oxygen and nutrient supply due to relative regional hypoperfusion. Watershed territories have been consistently considered at increased risk due to long perforating vessel high functional vulnerability. This hemodynamic regional vulnerability is well known in major vessel occlusions (e.g. in internal carotid artery occlusion with an inefficient circle of Willis compensation, in moya-moya disease, etc.). Similarly, watershed territories have been considered at risk in severe anemia, whenever the increase in blood perfusion did not compensate for low hemoglobin levels [11]. Vascular-like lesions in these regions (mostly located in the centrum semiovale deep white matter [12, 13]) have been therefore considered as the hallmark of anemia-related brain damage and several studies on other genetically determined hemoglobinopathies such as sickle cell disease have shown watershed silent infarcts in up to 40% of pediatric patients [13].

Recently, Choi and colleagues have shown decreased white matter volume and microstructural integrity in the watershed regions in a large sample of anemia patients (including beta-thalassemia subjects) [8, 9], supporting the hypothesis of an increased regional vulnerability to hypoperfusion in conditions of anemia. Even though white matter volume changes were not confirmed in a whole brain voxel-based analysis on our beta-thalassemia population [14], this evidence was considered highly interesting and prompted further perfusion analyses on our study population. The analyses showed an unexpected finding in the watershed territories with relative hyperperfusion in comparison with healthy subjects instead of hypoperfusion, thus reversing, at least in beta-thalassemia, the pathogenetic hypothesis of white matter injury. Indeed, white matter is known to be vulnerable to both hypoperfusion and hyperperfusion [15]. Although the latter is less known, increased cerebral blood perfusion might be associated with blood–brain barrier breakdown, abnormal cerebrovascular reserve, increased blood flow velocities and subsequent parenchymal injury. Interestingly, restricting the analysis to the areas of relative hyperperfusion, decreased white matter volume was found also in our study population compared to healthy controls, suggesting not only that volume changes might be subtle, so that they emerge only with focused analysis, but also supporting a strict relationship between regional hemodynamics and white matter changes, that challenges in beta-thalassemia the pathogenic role of chronic impairment of oxygen and nutrients supply.

The fact that relative hyperperfusion in the watershed territories correlates to the severity of anemia might explain the white matter volume and connectivity decrease according to anemia severity [8, 9] as a consequence of enduring relative hyperperfusion instead of hypoperfusion. According to our study, the more the patient is anemic, the more perfused is his brain, the higher is the relative hyperperfusion in his supra and infratentorial watershed territories.

In addition, the linear regression analysis indicates that, in beta-thalassemia, perfusion values normalize with hemoglobin levels above 12 g/dL, i.e. at those ranges that are considered normal in the general population. This fact implicitly confirms that hemoglobin levels are the major determinant for perfusion changes in both TDT and NTDT patients, thus minimizing the role of other possible disease-related factors. However, keeping hemoglobin above 12 g/dl is not acceptable for the excess iron loading while levels above 9.5 g/dL seem to be sufficient to keep brain hyperperfusion changes at a non-significant level compared to healthy controls. Therefore, our findings reinforce the strict adoption of the recommendations from the Thalassemia International Federation setting the pretransfusion hemoglobin level at 9.5 g/dL, as the desirable target for protecting the brain while minimizing the iron poisoning of all the other organs (the brain appears to be protected from iron overload as shown in a previous study on this issue) [3]. 

Two main study findings appear controversial. Firstly, differently from relative hyperperfusion values, white matter volume reduction was independent from hemoglobin levels. Indeed, our analysis considered the hemoglobin level at the MRI scan, while volume changes likely result from a cumulative injury during hemoglobin level fluctuations in the patients’ life. According to this explanation, patient management should aim at keeping hemoglobin persistently above those levels that are associated to watershed areas relative hyperperfusion.

Besides, white matter density changes were correlated with cognitive deficits only in NTDT patients who are known to be cognitively less affected than TDT. Possibly, with increasing disease severity, other factors such as a higher rate of hospitalization, transfusion related complications etc. might have a major impact in cognition overcoming the effect of structural white matter changes.

## Limits

This is the first study investigating brain perfusion in a sufficiently large beta-thalassemia sample. We applied a non-invasive MRI technique, but there are some other techniques (e.g. contrast enhanced perfusion MRI, ^15^O-H_2_O positron emission tomography, multi-delay arterial spin labeling, etc.) that need to be applied to confirm and validate our findings.

Hemoglobin levels on the day of MRI were not available in healthy controls for ethical and cost reasons. As the sample was relatively large while the probability to have sudden asymptomatic abnormal levels of hemoglobin in asymptomatic subjects without history of anemia or polycythemia is rather low, we are confident that this aspect should not have had a significant impact on the study findings. Hypothesizing a high rate of low hemoglobin level in the healthy subgroup would imply that beta-thalassemia per se and not hemoglobin levels is the reason for a dramatic increased brain perfusion in patients. Hypothesizing a high rate of polycythemia in our control group is weird as the estimated prevalence of polycythemia in the general population is about 22 per 100,000 [16]. 

Our multicenter study included only Italian centers in order to centralize MRI examinations on a single scanner and minimize technical confounding factors. A multicenter study including other European and extra-European centers is required to validate our data in a multiracial and multi-environment setting.

## Conclusions

Relative hyperperfusion of supratentorial and infratentorial watershed territories seems to represent a hemodynamic hallmark of beta-thalassemia anemia shedding a new light on the pathogenesis of brain injury in hereditary anemias. A careful management of anemia severity might be crucial for preventing structural white matter changes and subsequent long term cognitive impairment.

## Data Availability

Data not shared to protect patients’ privacy as still used for ongoing research, but available upon request to the corresponding author in anonymized files.
